# Supplementary material for: Optimizing NiFe-Modified Graphite for Enhanced Catalytic Performance in Alkaline Water Electrolysis: Influence of Substrate Geometry and Catalyst Loading
Source: Molecules. 2024 Oct 8;29(19):4755. doi: 10.3390/molecules29194755 (PMC11482479; doi:10.3390/molecules29194755)
Supplement: Supplementary file 1 [file molecules-29-04755-s001.zip › molecules-3222954-supplementary.pdf]

Supplementary file

Optimizing NiFe-Modified Graphite for Enhanced Catalytic Performance in Alkaline Water Electrolysis: Influence of Substrate Geometry and Catalyst Loading

Mateusz Kuczyński [1], Tomasz Mikołajczyk [1], Bogusław Pierożyński *[1], Jakub Karczewski [2]

[1]Department of Chemistry, Faculty of Agriculture and Forestry, University of Warmia and Mazury in Olsztyn, Łódzki Square 4, 10-727 Olsztyn, Poland; mateusz.kuczynski@uwm.edu.pl (M.K.); tomasz.mikolajczyk@uwm.edu.pl (T.M.)
[2]Institute of Nanotechnology and Materials Engineering, Faculty of Applied Physics and Mathematics, Gdansk University of Technology ul. G. Narutowicza 11/12, Gdańsk, Poland jakub.karczewski@pg.edu.pl (J.K)
* Correspondence: boguslaw.pierozynski@uwm.edu.pl or bogpierozynski@yahoo.ca (B.P.);

Abstract:

The oxygen evolution reaction (OER) and hydrogen evolution reaction (HER) are critical processes in water splitting, yet achieving efficient performance with minimal overpotential remains a significant challenge. Although NiFe-based catalysts are widely studied, their performance can be further enhanced by optimizing the interaction between the catalyst and the substrate. Here, we present a detailed investigation of NiFe-modified graphite electrodes, comparing the effects of compressed and expanded graphite substrates on catalytic performance. Our study reveals that substrate geometry plays a pivotal role in catalyst distribution and activity, with expanded graphite facilitating more effective electron transfer and active site utilization. Additionally, we observe that increasing NiFe loading leads to only modest gains in performance due to catalyst agglomeration at higher loadings. The optimized NiFe-graphite composites exhibit superior stability and catalytic activity, achieving lower overpotentials and higher current densities, making them promising candidates for sustainable hydrogen production in alkaline electrolysis.

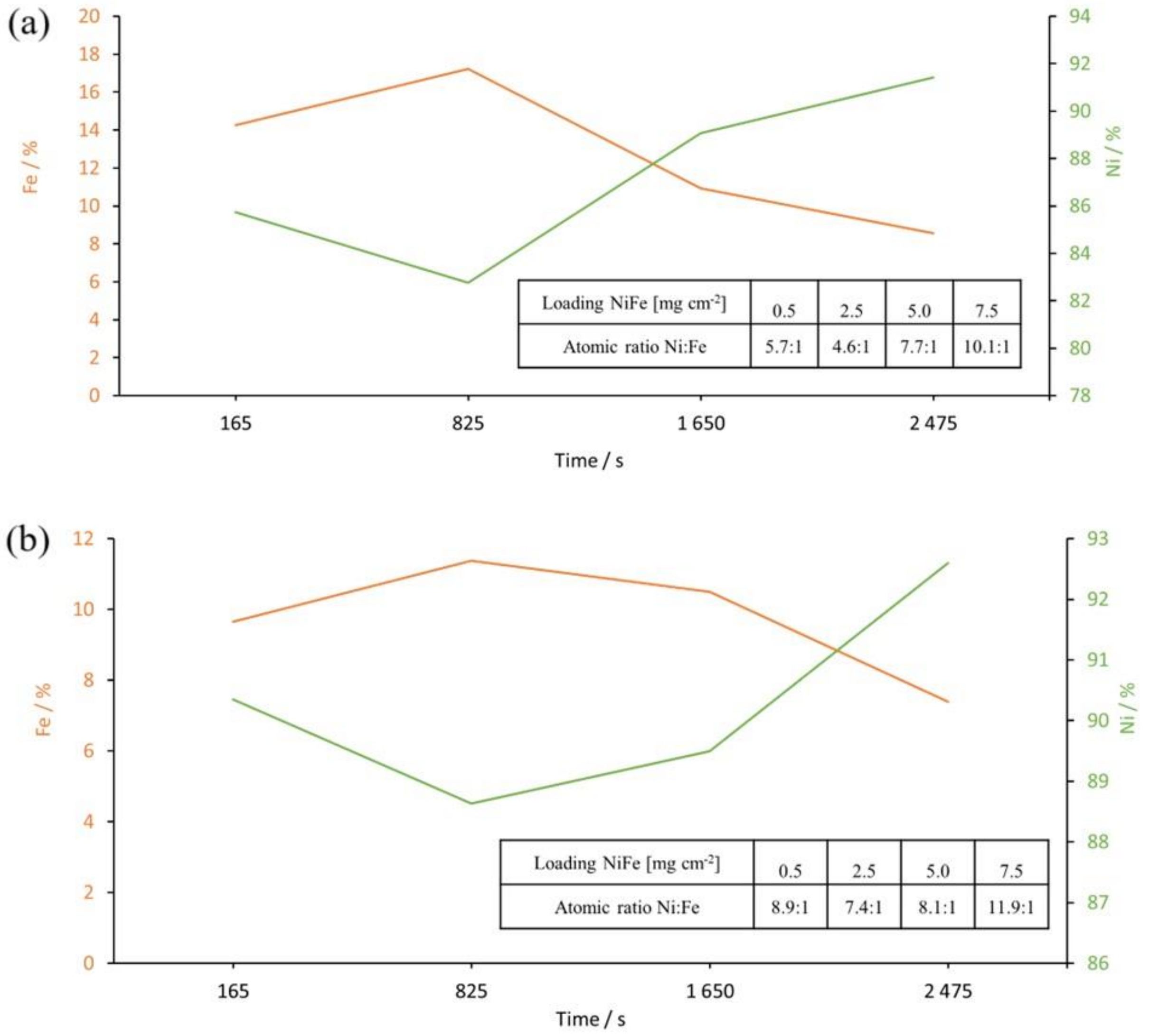

Figure S1. Elemental Fe and Ni content and ratio depending on the deposition time for expanded graphite (a) and compressed graphite (b) composites.

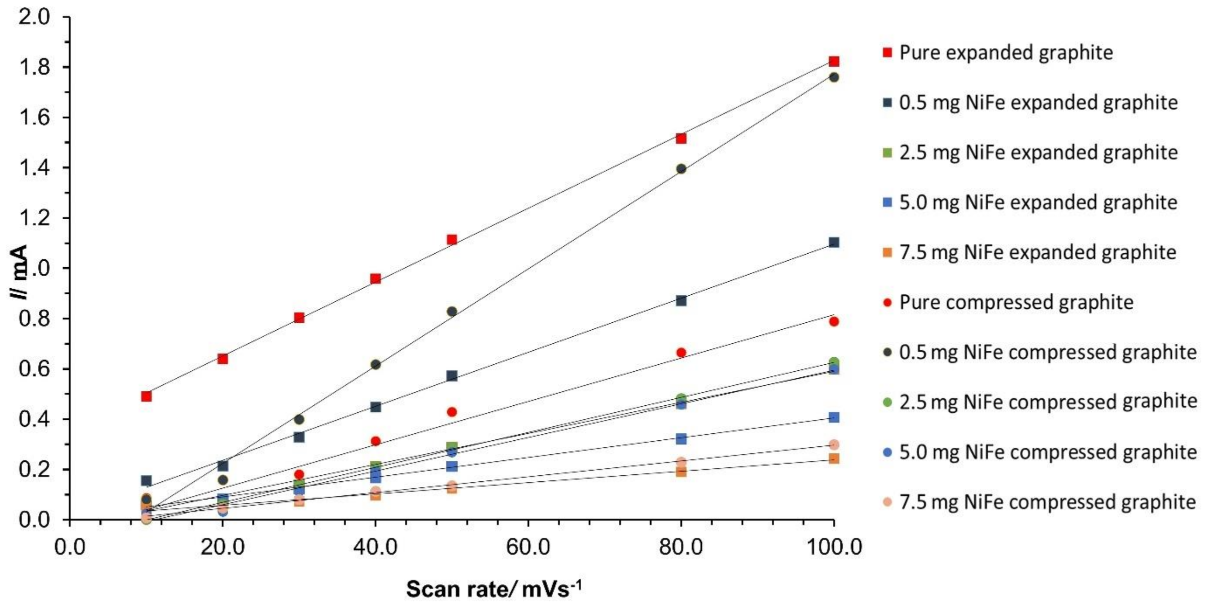

Figure S2. Average current measured at the potential of 0.7 V vs. RHE, plotted as a function of scan rate. The slope of the linear fit gives the double-layer capacitance.

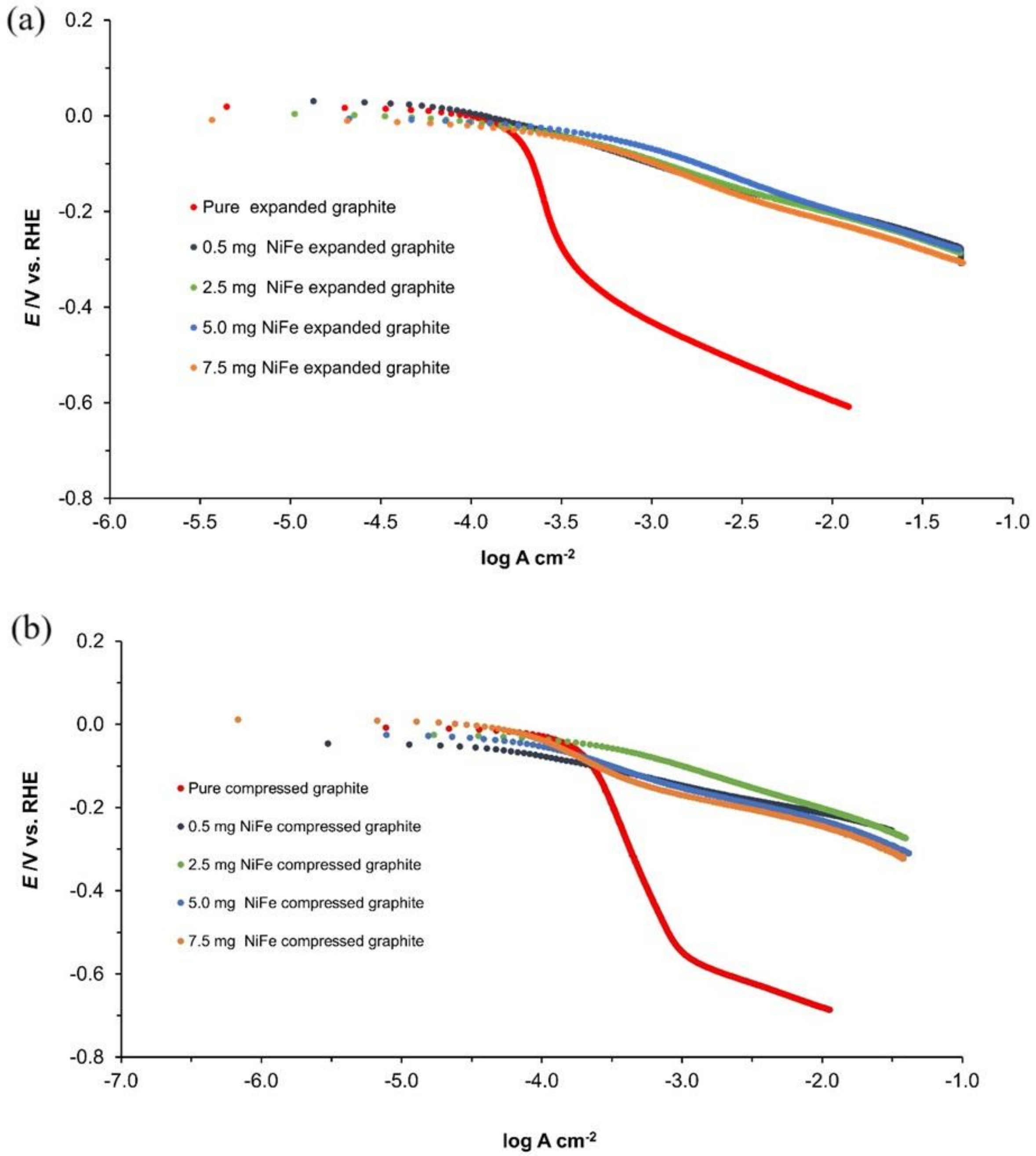

Figure S3. Quasi-potentiostatic cathodic polarisation curves for the HER, obtained at expanded graphite (a) and compressed graphite (b) electrodes, both unmodified and modified with various NiFe loadings in 1.0 M NaOH electrolyte. The curves were recorded at a scan rate of 0.5 mVs^{-1} (iR-corrected).

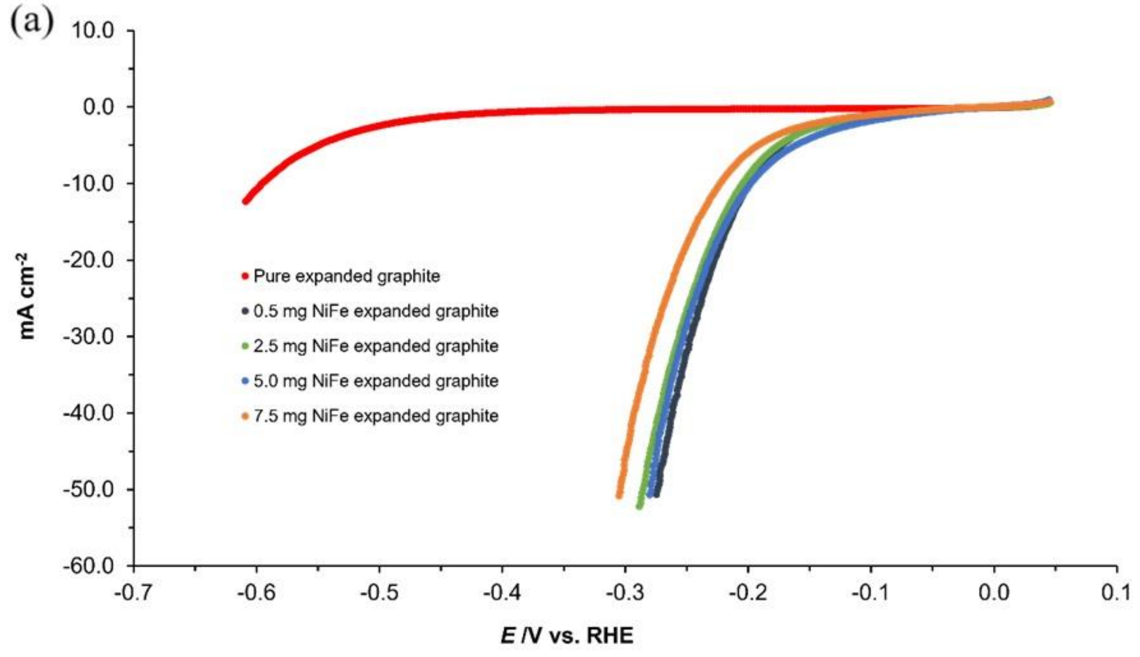

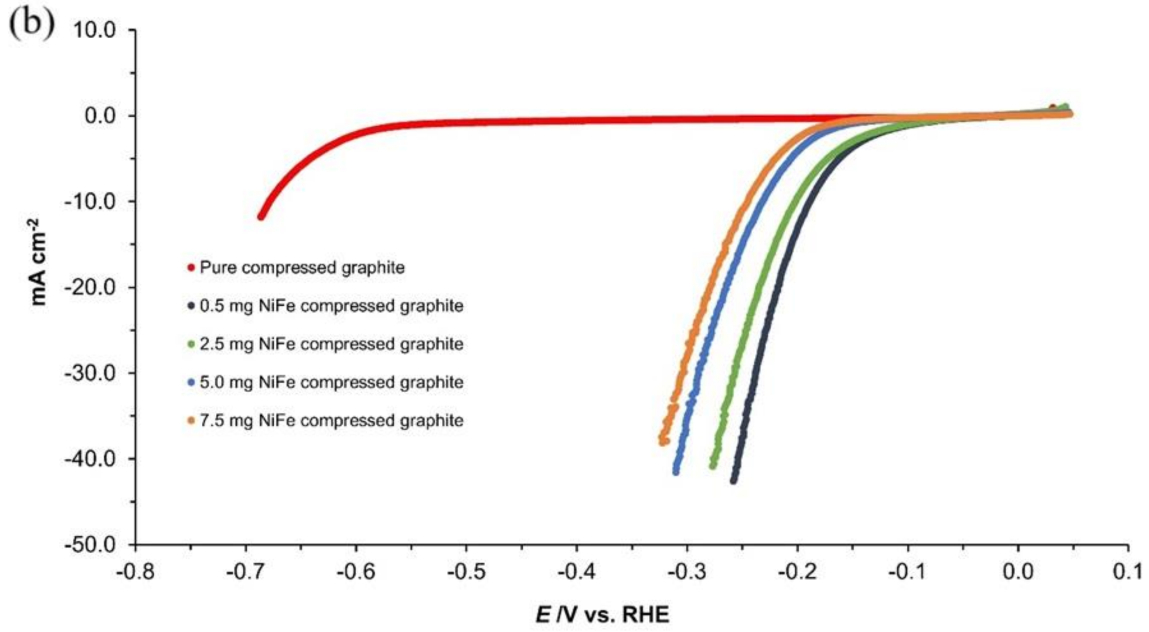

Figure S4. Linear Sweep Voltammetry (LSV) curves of expanded graphite (a) and compressed graphite (b) electrodes, both unmodified and modified with various NiFe loadings in 1.0 M NaOH solution, carried out with a scan rate of 0.5 mV s^{-1} for the HER (iR-corrected).

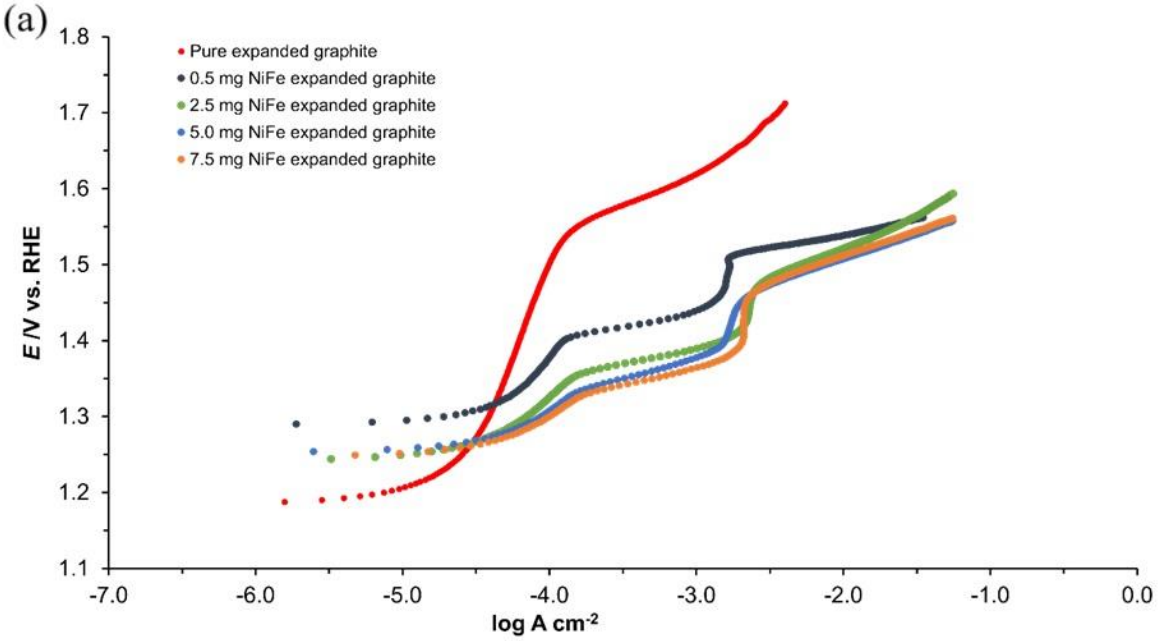

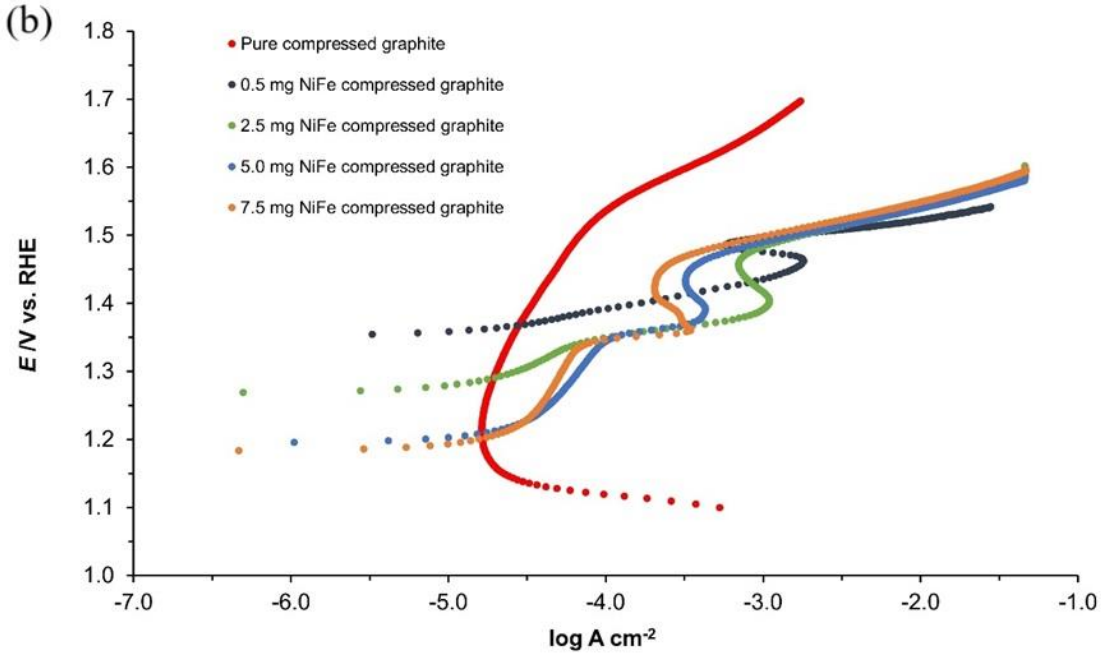

Figure S5. Quasi-potentiostatic cathodic polarisation curves for the OER, obtained at expanded graphite (a) and compressed graphite (b) electrodes, both unmodified and modified with various NiFe loadings in 1.0 M NaOH electrolyte. The curves were recorded at a scan rate of 0.5 mVs^{-1} (iR-corrected).

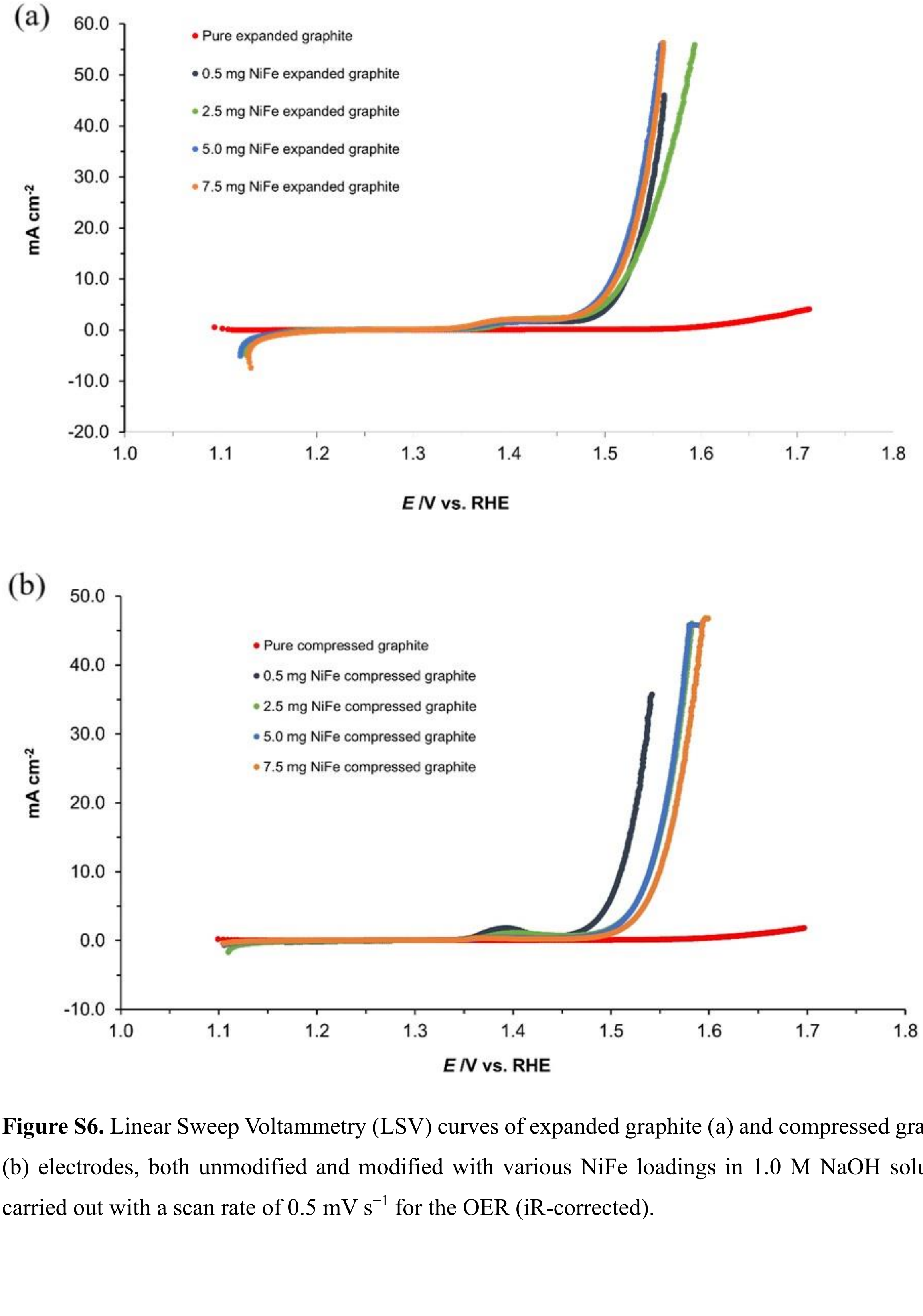

Figure S6. Linear Sweep Voltammetry (LSV) curves of expanded graphite (a) and compressed graphite (b) electrodes, both unmodified and modified with various NiFe loadings in 1.0 M NaOH solution, carried out with a scan rate of 0.5 mV s^{-1} for the OER (iR-corrected).

Table S1. Composition of electrodeposition baths and process parameters to prepare NiFe alloy deposits.

**Reagents**	**Concentration (M)**	**System and process parameters**
$NiSO_4 \times 6\ H_2O$ (99.0%, Sigma Aldrich, Saint Louis, USA)	0.48	Anode: Pt foil<br>Cathode: expanded graphite and compressed graphite<br>Temperature: 313 K<br>Deposition time:<br>0.5 mg 165 s<br>2.5 mg 825 s<br>5.0 mg 1651 s<br>7.5 mg 2477 s<br>Current-density: 1 A dm^{-2}<br>Solution pH: 3.0
$NiCl_2 \times 6\ H_2O$ (98%, POCH, Gliwice, Poland)	0.58	
H_3BO_3 (>99.5%, POCH, Gliwice, Poland)	0.73	
$FeSO_4 \times 7\ H_2O$ (99%, AKTYN, Suchy Las, Poland)	0.07	

Table S2. AFM derived the average increase of the surface area for NiFe-modified graphite electrodes regarding all scan sizes.

	expanded graphite					compressed graphite				
Loading [mg cm^{-2}]	0	0.5	2.5	5.0	7.5	0	0.5	2.5	5.0	7.5
Increase of surface area [%]	2.8	21.9	21.7	45.5	50.5	20.9	59.7	54.4	63.6	22.4

Table S3. Electrochemical impedance parameters for the HER obtained on expanded graphite and compressed graphite electrodes, both unmodified and modified with various NiFe loadings in 0.1 M NaOH supporting solution. The results obtained here were recorded by fitting the CPE-modified Randles equivalent circuit (see Figure 6b–d) to the experimentally obtained impedance data (reproducibility usually below 10%, $\chi^2 = 1.56 \times 10^{-6}$ to 1.74×10^{-5}).

E/mV	R_p/Ω cm^2	C_p/µF cm^{-2}	R_{ct}/Ω cm^2	C_{dl}/µF cm^{-2}
**Unmodified expanded graphite**				
-50	17.3 ± 1.1	3495 ± 346	-	7999 ± 380
-100	16.1 ± 1.1	3274 ± 379	-	8656 ± 416
-150	15.7 ± 1.1	3315 ± 39	-	8930 ± 436
-200	15.1 ± 1.2	3286± 398	-	9253 ± 436
-250	15.6 ± 1.4	3703 ± 401	-	8971 ± 440
-300	14.4 ± 0.9	3400 ± 422	-	9520 ± 464
-350	21.5 ± 0.8	6166 ± 180	-	6444 ± 195
-400	28.3 ± 1.1	7863 ± 143	-	4693 ± 152
-450	10.5 ± 0.4	4447 ± 320	396.2 ± 7.4	12351 ± 35
-500	11.3 ± 0.4	4659 ± 990	169.5 ± 2.1	12194 ± 64
-550	9.8 ± 0.4	3711 ± 353	90.9 ± 1.1	12310 ± 92
-600	9.5 ± 0.6	3242 ± 424	42.0 ± 1.1	12703 ± 218
-700	9.4 ± 0.6	3412 ± 509	35.4 ± 0.9	11843 ± 244
**NiFe 0.5 mg cm^{-2}**				
-50	51.2 ± 3.3	11879 ± 237	123.1 ± 0.7	13277 ± 185
-100	-	-	87.2 ± 0.4	8825 ± 43
-150	-	-	49.1 ± 0.1	7700 ± 37
-200	-	-	25.6 ± 0.1	6145 ± 55
-250	-	-	17.1 ± 0.1	5375 ± 84
-300	-	-	12.4 ± 0.1	4236 ± 93
-350	-	-	9.2 ± 0.1	3821 ± 107
-400	-	-	7.4 ± 0.1	3855 ± 177
-450	-	-	6.1 ± 0.1	3474 ± 241
-500	-	-	5.1 ± 0.1	2885 ± 191
**NiFe 2.5 mg cm^{-2}**				
-50	22.2 ± 3.3	9257 ± 436	123.7 ± 1.4	10673 ± 173
-100	13.6 ± 1.1	7418 ± 192	89.9 ± 0.3	8284 ± 32

-150	15.4 ± 2.8	137687 ± 28	50.6 ± 0.3	7557 ± 83
-200	14.1 ± 2.1	6183 ± 215	27.9 ± 0.1	5689 ± 114
-250	-	-	15.8 ± 0.1	4241 ± 80
-300	-	-	10.1 ± 0.1	2975 ± 53
-350	-	-	7.5 ± 0.3	2378 ± 38
-400	-	-	5.8 ± 0.1	2077 ± 45
-450	-	-	4.7 ± 0.1	1948 ± 43
-500	-	-	3.9 ± 0.1	1777 ± 48
**NiFe 5.0 mg cm^{-2}**				
-50	170.6 ± 0.8	3970 ± 155	178.0 ± 24.6	3022 ± 168
-100	110.9 ± 0.2	2523 ± 32	139.8 ± 4.9	3671 ± 68
-150	-	-	55.3 ± 0.1	1430 ± 8
-200	-	-	30.1 ± 0.1	1241 ± 1
-250	-	-	14.5 ± 0.1	1067 ± 21
-300	-	-	8.9 ± 0.1	927 ± 26
-350	-	-	7.8 ± 0.1	884 ± 31
-400	-	-	5.4 ± 0.1	907 ± 47
-450	-	-	4.4 ± 0.1	848 ± 56
-500	-	-	3.4 ± 0.1	954 ± 61
**NiFe 7.5 mg cm^{-2}**				
-50	238.0 ± 0.9	2205 ± 37	276.5 ± 4.6	1360 ± 16
-100	123.4 ± 0.5	1368 ± 154	218.7 ± 158.4	1874 ± 604
-150	-	-	57.8 ± 0.1	648 ± 5
-200	-	-	26.2 ± 0.1	554 ± 11
-250	-	-	16.0 ± 0.1	506 ± 17
-300	-	-	11.1 ± 0.1	526 ± 23
-350	-	-	8.7 ± 0.1	517 ± 24
-400	-	-	6.9 ± 0.1	491 ± 26
-450	-	-	5.7 ± 0.1	464 ± 27
-500	-	-	5.2 ± 0.1	521 ± 25
**Unmodified compressed graphite**				
-50	4.3 ± 0.2	9783 ± 688	2162.9 ± 19.1	2766 ± 4
-100	4.2 ± 0.2	6497 ± 422	2342.2 ± 23.3	2780 ± 5
-150	5.1 ± 0.1	7562 ± 258	1976.6 ± 15.7	2845 ± 3
-200	5.4 ± 0.3	8481 ± 850	1689.5 ± 11.5	2968 ± 3

-250	5.5 ± 0.3	9000 ± 955	1383.8 ± 8.8	3093 ± 3
-300	4.6 ± 0.2	8402 ± 323	1110.3 ± 8.2	3238 ± 4
-350	3.4 ± 0.2	7364 ± 414	877.5 ± 6.6	3360 ± 6
-400	2.8 ± 0.2	7332 ± 497	647.5 ± 5.9	3507 ± 8
-450	1.4 ± 0.2	4066 ± 772	494.3 ± 5.9	3763 ± 16
-500	0.8 ± 0.1	4340± 811	340.8 ± 4.0	4160 ± 19
-550	30.8 ± 2.7	8472 ± 363	315.2 ± 8.6	7320 ± 285
-600	26.0 ± 5.6	7271 ± 664	168.1 ± 10.7	9889 ± 1336
-650	38.2 ± 3.6	7097 ± 200	140.8 ± 6.9	20992 ± 2072
-700	22.4 ± 6.8	5242 ± 464	51.7 ± 5.4	40964 ± 3549
**NiFe 0.5 mg cm^{-2}**				
-50	3.6 ± 0.8	3923 ± 1150	39.1 ± 1.9	9741 ± 110
-100	5.5 ± 1.4	5299 ± 1222	43.9 ± 0.2	10283 ± 194
-150	-	-	30.7 ± 0.1	7601 ± 38
-200	-	-	20.3 ± 0.1	6816 ± 55
-250	-	-	13.8 ± 0.1	6149 ± 84
-300	-	-	10.5 ± 0.1	5811 ± 117
-350	-	-	8.1 ± 0.1	5463 ± 142
-400	-	-	7.1 ± 0.1	6277 ± 153
-450	-	-	5.7 ± 0.1	5501 ± 172
-500	-	-	4.9 ± 0.1	5439 ± 183
**NiFe 2.5 mg cm^{-2}**				
-50	-	-	97.5 ± 0.9	3154 ± 45
-100	-	-	76.9 ± 0.3	2795 ± 21
-150	-	-	46.9 ± 0.1	2349 ± 19
-200	-	-	28.4 ± 0.1	2040 ± 24
-250	-	-	19.1 ± 0.1	1876 ± 37
-300	-	-	13.5 ± 0.1	1908 ± 66
-350	-	-	10.4 ± 0.1	1814 ± 73
-400	-	-	8.4 ± 0.1	2081 ± 137
-450	-	-	6.7 ± 0.1	1457 ± 99
-500	-	-	5.9 ± 0.2	194 ± 291
**NiFe 5.0 mg cm^{-2}**				
-50	-	-	318.7 ± 1.9	976 ± 7
-100	-	-	200.9 ± 0.3	821 ± 2

-150	-	-	83.1 ± 0.2	670 ± 6
-200	-	-	33.2 ± 0.2	590 ± 16
-250	-	-	18.0 ± 0.1	55 ± 21
-300	-	-	12.7 ± 0.1	564 ± 27
-350	-	-	7.7 ± 0.3	34889 ± 1431
-400	-	-	9.5 ± 0.2	727 ± 58
-450	-	-	8.2 ± 0.9	676 ± 60
-500	-	-	6.7 ± 0.2	630 ± 75
**NiFe 7.5 mg cm^{-2}**				
-50	-	-	362.8 ± 3.8	841 ± 9
-100	-	-	362.8 ± 3.7	841 ± 9
-150	-	-	74.5 ± 0.2	550 ± 5
-200	-	-	29.2 ± 0.2	493 ± 14
-250	-	-	15.9 ± 0.1	419 ± 19
-300	-	-	11.9 ± 0.1	382 ± 20
-350	-	-	9.1 ± 0.1	454 ± 25
-400	-	-	7.2 ± 0.1	466 ± 46
-450	-	-	5.7 ± 0.1	417 ± 25
-500	-	-	5.1 ± 0.1	392 ± 33

Table S4. Electrochemical parameters for the OER obtained on expanded graphite and compressed graphite electrodes, both unmodified and modified with various NiFe loading levels in 0.1 M NaOH supporting solution. The results obtained here were recorded by fitting the CPE-modified Randles equivalent circuits (see Figure 6b–d) to the experimentally obtained impedance data (reproducibility usually below 10%, $\chi^2 = 1.56 \times 10^{-6}$ to 1.74×10^{-5}).

E/mV	R_p/Ω cm^2	C_p/µF cm^{-2}	R_{ct}/Ω cm^2	C_{dl}/µF cm^{-2}
**Unmodified expanded graphite**				
1300	72.5 ± 4.2	1427 ± 129	-	4762 ± 153
1350	54.5 ± 2.2	2504 ± 130	-	4853 ± 157
1400	50.2 ± 2.2	2302 ± 144	-	5379 ± 173
1450	50.1 ± 2.1	2358 ± 138	-	5539 ± 168
1500	54.8 ± 2.2	2777 ± 137	-	5189 ± 168
1550	59.7 ± 2.1	3129 ± 117	-	4932 ± 145
1600	44.9 ± 3.5	3103 ± 247	523.2 ± 80.7	8816 ± 108
1650	37.3 ± 1.2	2070 ± 96	142.7 ± 2.2	9505 ± 131
1700	41.7 ± 2.0	12413 ± 660	51.9 ± 1.9	1731 ± 87
1750	48.7 ± 3.7	1607 ± 690	36.2 ± 2.3	15106 ± 4261
1800	36.4 ± 1.7	906 ± 27	53.0 ± 3.5	11069 ± 847
**NiFe 0.5 mg cm^{-2}**				
1300	35.3 ± 3.3	3606 ± 301	-	14140 ± 195
1350	47.7 ± 1.4	5645.3 ± 122.9	-	15061 ± 102
1400	-	-	351.9 ± 26.3	31733 ± 188
1450	-	-	315.8 ± 9.6	28116 ± 99
1500	-	-	41.1 ± 0.1	20773 ± 51
1550	-	-	15.4 ± 0.1	16731 ± 148
1600	-	-	8.6 ± 0.1	16106 ± 238
1650	-	-	6.1 ± 0.1	13978 ± 383
1700	-	-	4.7 ± 0.1	12167 ± 700
1750	-	-	4.3 ± 0.2	15606 ± 1567
1800	-	-	3.4 ± 0.2	19283 ± 2338
**NiFe 2.5 mg cm^{-2}**				
1300	33.0 ± 2.1	4241 ± 230	-	9763 ± 166
1350	51.9 ± 1.4	4801.8 ± 73.4	-	8521 ± 61
1400	-	-	2427.2 ± 469.4	27367 ± 81
1450	-	-	250.3 ± 4.9	47245 ± 79
1500	-	-	33.6 ± 0.1	45891 ± 160

1550	-	-	13.6 ± 0.1	43523 ± 310
1600	-	-	6.8 ± 0.1	44530 ± 873
1650	-	-	5.5 ± 0.1	58252 ± 2052
1700	-	-	3.8 ± 0.1	51473 ± 2756
1750	-	-	3.5 ± 0.3	65456 ± 6946
1800	-	-	2.2 ± 0.1	34172 ± 3782
**NiFe 5.0 mg cm^{-2}**				
1300	489.1 ± 15.4	2594 ± 18	-	4710 ± 148
1350	748.2 ± 24.1	6550 ± 29	-	25570 ± 3774
1400	-	-	874.4 ± 23.3	9843 ± 39
1450	-	-	360.9 ± 3.2	22408 ± 44
1500	-	-	47.1 ± 0.1	22100 ± 32
1550	-	-	16.1 ± 0.1	20592 ± 96
1600	-	-	8.5 ± 0.1	20093 ± 238
1650	-	-	4.9 ± 0.1	19533 ± 300
1700	-	-	3.6 ± 0.1	19606 ± 677
1750	-	-	3.6 ± 0.1	19560 ± 901
1800	-	-	2.8 ± 0.1	20131 ± 1323
**NiFe 7.5 mg cm^{-2}**				
1300	305.5 ± 7.8	2362 ± 15	-	4038 ± 54
1350	212.2 ± 21.2	3804 ± 51	-	3468 ± 105
1400	-	-	506.6 ± 15.5	18150 ± 89
1450	-	-	294.5 ± 4.4	21000 ± 46
1500	-	-	51.3 ± 0.1	21468 ± 42
1550	-	-	14.93 ± 0.1	21802 ± 100
1600	-	-	7.7 ± 0.1	21551 ± 143
1650	-	-	5.1 ± 0.1	20905 ± 214
1700	-	-	3.8 ± 0.1	20721 ± 213
1750	-	-	3.1 ± 0.1	19275 ± 320
1800	-	-	2.5 ± 0.2	18765 ± 589
**Unmodified compressed graphite**				
1300	-	-	-	1607 ± 6
1350	-	-	-	1631 ± 7
1400	-	-	-	1657 ± 7
1450	-	-	-	1731 ± 8
1500	-	-	6020.1 ± 227.8	1703 ± 6

1550	-	-	2847.8 ± 59.1	1679 ± 7
1600	-	-	942.9 ± 9.7	1651 ± 9
1650	-	-	343.2 ± 2.3	1617 ± 13
1700	-	-	187.6 ± 1.1	1505 ± 16
1750	-	-	131.8 ± 0.6	1441 ± 17
1800	-	-	101.0 ± 0.5	1498 ± 19
**NiFe 0.5 mg cm^{-2}**				
1300	46.5 ± 6.2	11402 ± 303	-	3527 ± 314
1350	12.3 ± 0.6	9835 ± 219	-	11908 ± 212
1400	2.1 ± 0.2	31534 ± 2106	-	30076 ± 2104
1450	0.5 ± 0.1	18966 ± 4915	102.1 ± 2.3	70962 ± 194
1500	0.5 ± 0.1	62836 ± 8838	32.2 ± 0.2	66025 ± 188
1550	-	-	14.9 ± 0.1	63601 ± 312
1600	-	-	9.5 ± 0.1	63908 ± 453
1650	-	-	7.1 ± 0.1	66238 ± 696
1700	-	-	5.6 ± 0.1	65691 ± 1141
1750	-	-	4.6 ± 0.1	67363 ± 1412
1800	-	-	4.3 ± 0.1	67769 ± 2389
**NiFe 2.5 mg cm^{-2}**				
1300	248.2 ± 53.6	7532 ± 279	-	-
1350	43.6 ± 3.1	5230 ± 300	-	-
1400	8.9 ± 0.9	6037 ± 788	-	-
1450	-	-	197.2 ± 8.9	2858 ± 374
1500	-	-	34.1 ± 0.1	10430 ± 335
1550	-	-	13.7 ± 0.1	28787 ± 596
1600	-	-	8.3 ± 0.1	50340 ± 178
1650	-	-	6.1 ± 0.1	50214 ± 96
1700	-	-	4.6 ± 0.1	50635 ± 131
1750	-	-	3.9 ± 0.1	51925 ± 330
1800	-	-	3.4 ± 0.1	52233 ± 462
**NiFe 5.0 mg cm^{-2}**				
1300	397.5 ± 17	3719 ± 28	-	3395 ± 110
1350	128.8 ± 23.5	5777 ± 81	-	3055 ± 90
1400	132.9 ± 9.8	30423 ± 303	-	15350 ± 45
1450	-	-	242.6 ± 2.8	23902 ± 45
1500	-	-	32.8 ± 0.1	26630 ± 93

1550	-	-	12.1 ± 0.1	28189 ± 196
1600	-	-	7.1 ± 0.1	29546 ± 359
1650	-	-	5.1 ± 0.1	32239 ± 419
1700	-	-	3.9 ± 0.1	33912 ± 718
1750	-	-	3.2 ± 0.1	31354 ± 1207
1800	-	-	2.5 ± 0.1	30010 ± 991
**NiFe 7.5 mg cm^{-2}**				
1300	-	-	-	2826 ± 11
1350	-	-	-	4044 ± 13
1400	-	-	-	26412 ± 133
1450	-	-	791.1 ± 20.5	17593 ± 31
1500	-	-	65.3 ± 0.3	19398 ± 64
1550	-	-	16.4 ± 0.1	20678 ± 134
1600	-	-	8.3 ± 0.1	21559 ± 456
1650	-	-	5.5 ± 0.1	21785 ± 807
1700	-	-	4.2 ± 0.1	24633 ± 1576
1750	-	-	3.6 ± 0.2	27398 ± 2997
1800	-	-	2.4 ± 0.1	16191 ± 2277
